# A Disintegrin and Metalloproteinase—Control Elements in Infectious Diseases

**DOI:** 10.3389/fcvm.2020.608281

**Published:** 2020-12-16

**Authors:** Ahmad Aljohmani, Daniela Yildiz

**Affiliations:** Institute of Experimental and Clinical Pharmacology and Toxicology, PZMS, ZHMB, Saarland University, Homburg, Germany

**Keywords:** metalloproteinase, infection, regulated intramembrane proteolysis, SARS – CoV, cardiovascular

## Abstract

Despite recent advances in treatment strategies, infectious diseases are still under the leading causes of death worldwide. Although the activation of the inflammatory cascade is one prerequisite of defense, persistent and exuberant immune response, however, may lead to chronicity of inflammation predisposing to a temporal or permanent tissue damage not only of the site of infection but also among different body organs. The initial response to invading pathogens is mediated by the recognition through various pattern-recognition receptors along with cellular engulfment resulting in a coordinated release of soluble effector molecules and cytokines aiming to terminate the external stimuli. Members of the ‘a disintegrin and metalloproteinase’ (ADAM) family have the capability to proteolytically cleave transmembrane molecules close to the plasma membrane, a process called ectodomain shedding. In fact, in infectious diseases dysregulation of numerous ADAM substrates such as junction molecules (e.g., E-cadherin, VE-cadherin, JAM-A), adhesion molecules (e.g., ICAM-1, VCAM-1, L-selectin), and chemokines and cytokines (e.g., CXCL16, TNF-α) has been observed. The alpha-cleavage by ADAM proteases represents a rate limiting step for downstream regulated intramembrane proteolysis (RIPing) of several substrates, which influence cellular differentiation, cell signaling pathways and immune modulation. Both the substrates mentioned above and RIPing crucially contribute to a systematic damage in cardiovascular, endocrine, and/or gastrointestinal systems. This review will summarize the current knowledge of ADAM function and the subsequent RIPing in infectious diseases (e.g., pathogen recognition and clearance) and discuss the potential long-term effect on pathophysiological changes such as cardiovascular diseases.

## Introduction

Inflammation is a defensive process by which the immune system responds to an external or internal stimulus aiming to remove the injurious insult and initiate the healing process (1, 2). One major inflammatory insult is infection caused by viruses, prions, bacteria, fungi, and parasites. Besides the infectious particles themselves, their released toxins may cause severe disease (3).

The response to invading pathogens follows a well-defined cascade. The initial recognition of the pathogen occurs through germline-encoded pattern-recognition receptors (PRRs) such as the Toll-like receptors (TLRs), C-type lectin receptors, NOD-like receptors, retinoic acid-inducible gene I (RIG)-like receptors, cyclic GMP-AMP synthase and stimulator of interferon genes (cGAS_STING), and scavenger receptors which are not only expressed by immune cells but also non-immune/tissue cells such as endothelial and epithelial cells (4–6). The pathogens themselves provide pathogen-associated molecular pattern (PAMP) such as lipopolysaccharide, lipoteichoic acid, and flagellin as bacterial component as well as nucleic acids mostly associated with viral infection. Activation of the PRRs by PAMPs and the cellular uptake of the pathogens result in the release of danger molecules and cytokines such as interleukin-1β (IL-1β), IL-6, IL-18, and tumor necrosis factor-α (TNF-α) orchestrating the immune response and defense. Chemoattraction and activation of neutrophils, T cells, monocytes and dendritic cells promote antigen presentation, pathogen phagocytosis and immune modification (7, 8) allowing for pathogen clearance, resolution of inflammation, and initiation of tissue homeostasis restoration. However, uncontrolled inflammation and insufficient clearance may lead to chronic inflammation and permanent tissues damage in the worst case leading to organ failure and sepsis (9).

ADAMs belong to the class of metalloproteinases and consist of 34 members, in which 22 are well-described in human (10, 11). ADAMs are transmembrane molecules consisting of an N-terminal metalloproteinase pro-domain, followed by a disintegrin domain, an epidermal growth factor (EGF)-like domain, a cysteine-rich domain, a transmembrane domain and a cytoplasmic domain (12). As the EGF-like domain is missing in ADAM10 and ADAM17, the membrane-proximal domain is often referred to as stalk region (12, 13). Twelve of the human ADAM members (ADAM8, 9, 10, 12, 15, 17, 19, 20, 21, 28, 30, and 33) are proteolytically active upon removal of the pro-domain that blocks the zinc atom (14). The proteolytic cleavage of transmembrane substrates close to the cell surface is referred to as ectodomain shedding and results in the release of the soluble ectodomains. These ectodomains may act as agonists (e.g., TNF-α and EGF), driving the inflammatory surrogate, or antagonists (e.g., TNFR and IL-1R), decreasing the inflammatory responsiveness and driving the resolution of inflammation (for review see (15, 16). The transmembrane and the cytoplasmic domain remain as cell-associated fragment and can be further processed by regulated intramembrane proteolysis (RIPing) through, e.g., γ-secretases (17), as a downstream event. The resulting C-terminal intracellular domains (ICDs) fulfill divergent functions, e.g., acting as endogenous inhibitor [e.g., syndecans (18)] or transcription factor [e.g., Notch (19)]. Regulation of ADAMs occurs on the posttranslational rather than transcriptional level. Posttranslational regulation includes the cleavage of the inhibiting pro-domain by furin (e.g., ADAM9, 10, and 17) and autocatalytic activation (e.g., ADAM8), the change in cellular distribution, the release from intracellular stores, interaction with partners for cellular trafficking and signaling as well as conformational changes (15). Dysregulation of the proteolytic activity of ADAMs is a core factor in a number of pathologies such as cardiovascular disease, asthma, cancer, inflammation, and neurodegenerative disease (20–24). Although the general function of ADAMs in inflammatory diseases is well-described, their specific contribution in infectious diseases, especially *in vivo* (**Table 2**) is only partly understood. This review will summarize the current knowledge of ADAMs' function in infectious diseases addressing pathogen recognition and entrance, toxin handling, phagocytosis and clearance, cytokine release, leukocyte recruitment, resolution of inflammation and repair/regeneration, as well as local and systemic changes (Table 1). The RIPing may influence further signaling pathways leading to immune modulation. Both ADAM function and the subsequent RIPing may lead to long-term effects on pathophysiological changes causing, e.g., cardiovascular diseases.

**Table 1:** Function of ADAM proteases in infection.

**Step**	**Protease**	**Substrate**	**Effect of shedding or non-proteolytical event**	**References**
Pattern recognition and entrance	ADAM9		EMCV entry receptor	(25, 26)
	ADAM10	CXCL16	Membrane bound scavenger receptor vs. soluble chemoattractant	(27, 28)
		TLR4	Reduced LPS responsiveness	(29)
			HIV nuclear entry and replication by ICD interaction	(30)
		TIM-1	Change in Zaire Ebolavirus recognition	(31)
	ADAM17	TNF-α	Enhanced bacterial uptake	(32)
		Gp96	Protection against *C. trachomatis* reinfection	(33)
		TNFR-1	Enhanced infection with *C. trachomatis*	(34)
			HPV entry through ERK1/2 activation	(35)
		CD16	Persistent HCV infection	(36)
		MICA	Escape of immune recognition by CMV	(37)
		ACE2	Reduced entrance of SARS-CoV2	(38)
		Syndecan-1	Change in HIV-1 attachment	(39)
Toxin handling	ADAM10		Receptor for *S. aureus* α-hemolysin (Hla)	(40)
Phagocytosis	ADAM10	CD163	Opsonization and enhanced phagocytosis	(41)
	ADAM17	TNF-α	Change in phagocytic capacity	(32)
		L-selectin	Enhanced clearance of *K. pneumoniae* by neutrophils	(42)
		CD163	Enhanced phagocytosis of *S. aureus*	(41, 43)
Mediator release and cell polarization	ADAM10	Notch	M1 macrophage and T_H_2 polarization	(44, 45)
	ADAM17	IL-6R	Trans-signaling and CCL-2 release	(46)
		EGFR ligands	IL-1 secretion by epithelial cells	(47)
		TNF-α	Inflammatory cytokine release	(48)
Cell recruitment	ADAM10		Enhanced inflammatory cell recruitment by integrin signaling	(49)
	ADAM17	L-selectin	Time-dependent leukocyte recruitment and improved survival in bacterial infection; clonal expansion of cytotoxic T cells in virus infection	(49–52)
Tissue damage, resolution of inflammation and systemic changes	ADAM9		Non-apoptotic cell death upon HIV infection	(53)
	ADAM10	CD46	Restriction of complement activation	(54)
		TIM-1/4	Apoptotic cell clearance	(30)
		FasL	Reduced killing capacity of T cells	(55)
		RAGE	Enhanced pathogenesis upon *P. aeruginosa* infection	(56)
		HB-EGF	Mucin overproduction and obstruction	(57)
		BTC	Cell apoptosis upon EPEC infection	(58)
		E-cadherin	Disruption of epithelial integrity, metastasis	(59)
		VE-cadherin	Disruption of endothelial integrity	(60)
	ADAM17	TNF-α	Endotoxic shock, T cell lethality, increase of MMP expression	(61–64).
		L-selectin	HIV release from central memory CD4^+^ T cells	(65)
		IL-6R	Systemic response and increase in lethality	(64)
		HB-EGF	Ulcus formation upon infection with *H. pylori*	(66)
		TNFRI/II	Reduced inflammatory burden	(67)
		TNFRI/II	CMV persistence	(68)
		ICAM	Leukocyte recruitment and endothelial damage	(69)
		VCAM	Leukocyte recruitment and endothelial damage	(69)
		syndecan-1	Systemic spread of bacteria	(70)
		ACE2	Arterial fibrillation and heart failure in SARS-Cov2 infection	(71, 72)

*ADAM proteases shed many general mediators of inflammation like TNFα, IL-1R, and IL-6R and repair/regeneration like growth factors [for review see (15)]. This table only summarizes (non)proteolytical functions, which have been mechanistically studied in infectious diseases with a direct link to ADAM proteases, e.g., genetic knockout or pharmacological inhibition*.

## ADAM10

***Pathogen recognition:*** Pathogen recognition is the first step of infection sensing. Cleavage of PRRs may cause [1] signaling initiation, [2] limitation of receptor-pathogen interaction, or [3] release of soluble scavenger receptor with opsonizing or antagonistic function. In the case of CD163, a scavenger receptor for both Gram-positive and Gram-negative bacteria (83), *Staphylococcus aureus* (*S. aureus*) leads to ADAM10-dependent release of soluble CD163, which binds to fibronectin on the bacterial surface resulting in further opsonization and higher clearance by phagocytes (41). Under certain conditions such as 1,25-dihydroxyvitamin D3 treatment, ADAM10 is able to shed TLR4 thereby limiting the inflammatory response to LPS (29). The third PRR is the receptor for advanced glycation end products (RAGE). Together with its secreted isoform (esRAGE), ADAM10-cleaved RAGE (cRAGE) builds the soluble fraction (sRAGE) (84), which has been shown to exert a detrimental action in *Pseudomonas aeruginosa* (*P. aeruginosa*) infection (56). However, a subsequent decrease of mature ADAM10 indicates a strict regulation and a time-dependent action of ADAM10 during *P. aeruginosa* infection (85). Besides its action in bacterial entry, ADAM10 might also be involved in viral recognition. T cell immunoglobulin and mucin domain (TIM)-1 and 4 are shed by ADAM10 (30), of which TIM-1 was shown to act as the receptor for the Zaire Ebola virus (31). ***Pathogen entrance/phagocytosis and toxin handling:*** ADAM10 may not only influence the initial pathogen interaction via cleavage of PRRs but also through its action as receptor itself. ADAM10 functions as a receptor for the *S. aureus* α-hemolysin (Hla) resulting in toxicity even at a low concentration (40). It was further shown that the intracellular domain of ADAM10 facilitates nuclear entry and replication of HIV-1 (86). CXCR6, the receptor for CXCL16 was previously identified as the HIV-1 coreceptor Bonzo (87). CXCL16, which is cleaved by ADAM10, exists as transmembrane form, mediating bacterial adhesion and phagocytosis, and soluble ectodomain with chemotactic function (27, 28, 88). ***Cell recruitment and polarization:*** ADAM10 is not only essential for the general recruitment of inflammatory cells (49), but also for the development of specific T cell responses. One crucial substrate of ADAM10, indicated by the lethality of ADAM10 knockout mice (81), is Notch which has divergent immune modulatory functions in infection. Upon infection with *Listeria monocytogenes* (*L. monocytogenes*), Notch downstream signaling results in pro-inflammatory polarization of macrophages to the classical M1 subtype (44). However, in allergic asthma induced by *Alternaria alternata* challenge, the T helper cell type 2 response was reduced upon Notch deficiency (45). Notch cleavage may also regulate invasiveness and epithelial to mesenchymal transition in epithelial cells infected with Epstein Barr virus (89). ***Tissue damage, resolution of inflammation, and systemic changes:*** There exist several evidences that ADAM10 does not only act in the initial phase of infection but is also involved in the development of tissue damage, the resolution of inflammation or development of systemic effects. E-cadherin and vascular endothelial (VE)-cadherin are junction molecules shed by ADAM10 in epithelial and endothelial cells, respectively (90, 91). Several pathogens, including *P. aeruginosa, S. aureus* and *Helicobacter pylori* (*H. pylori*) have been shown to induce cadherin shedding, thereby disrupting endothelial and epithelial barrier integrity leading to sepsis formation and persisting skin infection (59, 60, 73, 74, 92). Besides the mentioned molecules, shedding of growth factors such as heparin-binding EGF-like growth factor (HB-EGF) in epithelial cells may also contribute to persistent infection, e.g., through induction of mucin overproduction and obstruction (57). EGF receptor (EGFR) transactivation was further shown to induce epithelial cell apoptosis upon exposure to enteropathogenic *E. coli* (EPEC) (58). One essential step in the defense process and resolution of the concurrent infection is the removal of these apoptotic cells. Soluble TIM-1 and TIM-4 bind to phosphatidylserines on the surface of apoptotic cells, which could either prevent their engulfment or mediate phagocytosis by TIM-protein interaction (30). Furthermore, ADAM10 mediated shedding of Fas ligand (FasL) may reduce the killing capacity of T cells and their subsequent apoptosis, which could result in chronic inflammation (55). Additionally, ADAM10 sheds CD46 from apoptotic epithelial cells, which may reflect a strategy for restricted complement activation (54). As obvious, ADAM10 functions during different phases of infection (Tables 1, 2) and has to be tightly regulated via disease-specific mechanisms, which have to be taken into account when thinking about ADAM10 based treatment strategies.

**Table 2:** Infectious diseases in ADAM knockout animals.

**Transgene**	**Approaches**	**Model**	**Observation**	**References**
	Tamoxifen-inducible *Adam10^−/−^*	*Staphylococcus aureus* skin infection	Decrease in abscess size and protection against dermonecrotic lesions	(73, 74)
	*Adam17*^ex/ex^	*Listeria monocytogenes* infection	Diminished IL-6 receptor (IL-6R) trans-signaling in leukocyte subsets	(64)
Epithelial	SPC-rTA/TetO-Cre/*Adam10* ^flox/flox^	*Staphylococcus aureus*-Hla induced pneumonia	Reduction of epithelial gap formation and lethal pneumonia	(60)
Leukocytes	CD4-*Adam10^−/−^* LysM-*Adam10^−/−^*	*Listeria monocytogenes* infection	No contribution to IL-6R trans-signaling	(64)
	*Adam17 ^*flox*/*flox*^/Vav1-Cre*	Polymicrobial sepsis	Improved survival rate, reduced bacterial spread and pro-inflammatory cytokine secretion	(75, 76)
	*Adam17 ^*flox*/*flox*^/Vav1-Cre*	*E. coli* induced peritonitis	Improved survival rate, decreased bacterial load and neutrophil recruitment	(50, 77)
	*Adam17*^fl/fl^/*CD4-Cre*	*Listeria monocytogenes* infection	No influence on T cell response	(78)
	*Adam17*^−/−^ in CD8^+^ T cells	Influenza A (HA) infection	Reduced macrophage and neutrophil infiltration and decreased lethality	(79, 80)
	*F5*-Tg TCR*/LΔP* CD8^+^ T cells *(LΔP*: shedding resistant L-selectin)	Vaccinia virus infection	Lack of rapid clonal expansion of cytotoxic T cells	(52)
	*Adam10* ^DC−/−^	*Alternaria alternate* (allergic asthma)	Reduced eosinophils recruitment, serum IgG1 and IgE	(45)

*Mentioned are studies in which the animals were exposed to the pathogen itself and not only a specific PAMP like LPS. Notably, knockout of ADAM10 and ADAM17 results in pre-/perinatal lethality or death shortly after birth (81, 82)*.

## ADAM17

In addition to ADAM10, ADAM17 which is also known as tumor necrosis factor-α converting enzyme (TACE) (48) may play a divergent role during the different phases of bacterial, viral, and fungal infection. ***Pathogen recognition, entrance and phagocytosis*:** ADAM17 may influence the initial recognition and uptake of invading of pathogens by the cleavage of PRRs such as scavenger receptors and TLR2 and TLR4 (93, 94). However, it was recently shown that the limitation of bacterial uptake by ADAM17 in monocytes occurs in a cell-autonomous manner in parts dependent on TNF-α without significant changes in the surface expression of these receptors (32). Nevertheless, release of soluble CD163 from monocytes contributed to the recognition and phagocytosis of *S. aureus* (41, 43). In neutrophils, however, efficient bacterial clearance was dependent on ADAM17 activation and subsequent L-selectin shedding (42). Further, ADAM17 mediated shedding of the *Chlamydia trachomatis* receptor glucose regulated protein 96 (Gp96) protected against re-infection (33), whereas reduction of TNFRI expression on the cell surface facilitated the infection (34). With respect to viral infection ADAM17 facilitated the entry of the human papillomaviruses (HPV) (35), and led to downregulation of CD16 on natural killer (NK) cells during chronic hepatitis C virus (HCV) infection resulting in a lack of infection eradication (36). Furthermore, L-selectin was shown to support the preferential infection of central memory CD4^+^ T cells, with L-selectin shedding by ADAM17 being required for viral release (65). ***Cell recruitment and mediator release:*** ADAM17 sheds several junction and adhesion molecules like VCAM-1, ICAM-1 and JAM-A, which was shown to influence leukocyte recruitment and endothelial damage in both viral and sterile infection (69, 95, 96). Deficiency of ADAM17 in leukocytes resulted in enhanced recruitment of neutrophils to the site of infection, decreased bacterial load, and improved survival in both polymicrobial sepsis and peritonitis (50, 75, 77). Further investigations in models of sterile infection revealed a time-dependent effect of ADAM17 in neutrophil recruitment, which is dependent on L-selectin shedding at early time points (49–51). One essential step in the orchestration of the inflammatory response is the release of soluble mediators such as cytokines and growth factors. Stimulation of human airway epithelial cells with heat inactivated *S. aureus* and *P. aeruginosa* led to upregulation of ADAM17 surface expression and colocalization with IL-6R, initiating receptor trans-signaling and subsequent CCL-2 release (46). Similarly, ADAM17-mediated transactivation of EGFR was required for the upregulation of IL-1 in primary keratinocytes infected with *S. aureus* (47). The analysis of BAL samples from patients with community-acquired pneumonia showed that ADAM17 is involved in both the release of pro-inflammatory cytokines like TNF-α and IL-1β as well as their neutralizing soluble receptors (97). Furthermore, release of TNF-α by CD8^+^ T cells was essential for CXCL2 release and leukocyte recruitment upon influenza A infection (79). Additionally, L-selectin shedding promoted early clonal expansion of CD8^+^ T cell derived cytotoxic T cells in vaccinia virus infection (52). ***Tissue damage, resolution of inflammation, and systemic changes:*** Released mediators and their receptors do not only orchestrate the local immune response but are responsible for the systemic immune response resulting in either resolution of inflammation or in chronicity and damage of other organs. TNF-α shedding has been shown to be crucial for tissue damage and lethality in endotoxic shock, *S. pneumoniae* (serotype 3) caused meningitis, and *L. monocytogenes* infection, the latter one being further accompanied by IL-6R trans-signaling (61–64, 98). TNF-α is central during HIV infection and AIDS pathogenesis, in which a complex regulatory mechanism was observed. Nef induces both endocytosis of ADAM17, resulting in intracellular TNF-α cleavage, and ADAM17 release on extracellular vesicles, leading to release of TNF-α upon ingestion by PBMC, thereby decreasing the number of circulating CD4 lymphocytes (99, 100). Thus, the amount of circulating TNF-α has to be tightly regulated, e.g., through its soluble receptors TNFRI and TNFRII, which are shed by ADAM17 (67). On the one hand, enhanced shedding of TNFRs may reduce the inflammatory burden (80); on the other hand it may lead to reduced defense and persistent infection (68). A similar divergent function may apply for growth factor shedding. On the one hand, HB-EGF–mediated EGFR activation was shown to contribute to gastrointestinal ulcera and cancer formation upon *H. pylori* (66, 101, 102). On the other hand, TGF-α was required for β-defensins-3 (hBD-3) up-regulation upon *Candida albicans* infection (103). ***Escape mechanisms:*** ADAM17 does not only orchestrate the immune response but is also used by pathogens to escape the defense mechanisms. CMV promotes the shedding of MICA, an NK group 2D (NKG2D) receptor ligand, mimicking the tumor strategy in modulating immune recognition by NK cells and cytotoxic T cells (37). Furthermore, ADAM17 proteolytically cleaves the Ebola virus (EBOV) surface glycoprotein GP, which is known to block the antibodies responsible for virus neutralization (104). Thinking about treatment options, e.g., for polymicrobial sepsis (105), ADAM17 might be a suitable target in infectious diseases. However, due to its divergent functions (Tables 1, 2), as further addressed in the COVID-19 section, a substrate-specific, time-specific and site-specific therapeutic intervention has to be considered to achieve the best beneficial effect and reduction of severe side-effects.

## ADAM8

ADAM10 and ADAM17 as ubiquitously expressed proteases seem to play a central function in infectious diseases. Only a few studies have addressed the role of ADAM8. ADAM8 is less abundantly expressed with highest expression in leukocytes and carcinoma cells and seems to be dispensable for normal development and homeostasis (106, 107). In sterile inflammation caused by *E. coli* LPS, mainly leukocytic ADAM8 contributed to leukocyte recruitment in the acute phase of lung inflammation (108). ADAM8 protein expression was found to be upregulated during the formation of multinuclear giant cells by human salivary gland cells upon infection with human parainfluenza virus type 2 (HPIV2) (109). Further, macrophages of HIV-1 positive patients showed an upregulation of ADAM8 gene expression. In patients with periodontal diseases, which are mainly induced by bacteria and characterized by uncontrolled inflammation, a high protein expression ADAM8 was found in the gingival crevicular fluid, highly correlating with disease severity and destruction of the periodontal tissues (110). After non-surgical periodontal therapy, ADAM8 levels decreased and correlated with improvements in clinical parameters (111). Not only viral and bacterial infection may be influenced by ADAM8, but also infection by fungi. Transcriptional profiling of heart tissues mice infected with *Candida albicans* revealed an upregulation of ADAM8 gene expression, which was linked to cardiomyopathy and extracellular matrix remodeling (112). Thus, it seems feasible that ADAM8 plays a more important role in disease progression and chronicity vs. resolution of inflammation rather than in pathogen recognition and toxin handling.

## ADAM9

ADAM9 expression was clearly shown in the brain, the lung, the developing heart, and the retina as well as several cell types such as fibroblasts, neutrophils, and platelets (15). Although ADAM9 is less abundantly expressed than ADAM10 and ADAM17, it is involved in several steps of inflammation, including cytokine release, neutrophil activation, endothelial transmigration, and growth factor signaling (15, 113–115). Enhanced mRNA expression in the blood, which was mostly restricted to monocytes and neutrophils, has been found in patients with influenza caused pneumonia, respiratory syncytial virus (RSV) infection, active tuberculosis, and several other bacterial infections, whereas stimulation with PAMPs only marginally increased ADAM9 transcript abundance compared to healthy volunteers [for review see Rinchai et al. (116)]. Further, ADAM9 mRNA expression increased in cystic fibrosis patients during acute exacerbation, with its decline being an independent predictor of the antibiotic treatment response (117).

Mechanistic studies in infection highlight the relevance of ADAM9 during viral infection from pathogen entrance to systemic changes. ADAM9 was shown to be an important and specific factor for encephalomyocarditis virus (EMCV) entry, which was inhibited upon ADAM9 knockout in myeloid leukemia derived cells and HEK293T cells (25, 26). Interestingly, pharmacological inhibition of ADAM9 did not show any effect, suggesting a non-proteolytical function of ADAM9 as EMCV receptor (25). Furthermore, ADAM9 mRNA was highly expressed in central memory CD4 T cells during chronic HIV infection, which associated with movement to cell cycle phases G1 and S leading to non-apoptotic cell death (53). ADAM9 knockout protected against remodeling processes like elastin degradation and subsequent reduction in lung compliance in LPS-induced ALI (118). Thus, it seems feasible that ADAM9 is not only involved in the acute phase of infection but also in the decision between resolution of inflammation, chronicity, and systemic changes. Further evidences for systemic changes upon upregulation of ADAM9 are given by enhanced development of metastases during hepatitis B virus-related hepatocellular carcinoma (119).

## Catalytic Influence of Other ADAM Proteases on Infectious Diseases

Although 22 ADAM proteases have been reported in humans, only 12 display catalytic activity based on the presence of the zinc-dependent metalloproteinase domain (14). ADAM10 and ADAM17 together with ADAM8 and ADAM9 seem to be the most relevant ADAM proteases in infection; however, some studies point toward an additional influence of ADAM12, ADAM15, and ADAM33. Two ADAM12 genetic mutations (rs11244787 and rs1871054) led to an increased risk to transfer an infection with *Trypanosoma cruzi*, a parasitic euglenoids, from the mother to the newborn (120). Single nucleotide polymorphism (SNP) analysis revealed that an ADAM33 SNP was associated with a higher risk to develop severe RSV bronchiolitis with airway remodeling in premature infants (121, 122). In addition, children with acute infectious pneumonia of viral etiology showed an increased protein expression of ADAM28 and ADAM33 in the lung, which was correlated with the severity of infection and is likely to be involved in the chronicity and fibrosis development (123). So far, the contribution of ADAM15 has been only investigated in models of sterile infection. In both, LPS-induced ALI and sepsis ADAM15 deficiency protected against vascular endothelial barrier dysfunction, resulting in reduced neutrophil transmigration (124, 125). Further, *in vitro* studies revealed a more complex action of ADAM15 in inflammation and infection. Whereas, negative regulation of ADAM15 mediated by microRNA-147b significantly improved endothelial barrier dysfunction (126), ADAM15 dampened the TLR3 and TLR4 stimulated pro-inflammatory cytokine production (127). Thus, further studies are required to specify the stimulus- and cell-type specific contribution of single ADAM proteases in infectious diseases.

## Riping in Infectious Diseases

Not only the released ectodomains but also the remaining transmembrane and intracellular fragments may regulate the infectious response. Intramembrane cleaving proteases, such as the γ-secretase presenilin 1 (PSEN1), liberate the ICD which might act as antagonist [e.g., syndecan-1 and 4 (18)] or translocate to the nucleus acting as transcription factor [e.g., Notch (128, 129). Prerequisite to γ-secretase action is the previous cleavage by α-secretases (e.g., ADAM9, ADAM10, and ADAM17 (130)] or β-secretases [e.g., BACE (131)]. The function of the Notch ICD (NICD) as transcriptional regulator is widely accepted (132). The NICD translocates to the nucleus, where it binds to the DNA-binding protein RBP-J with subsequent recruitment of superenhancers initiating transcription of Notch downstream targets like Hes5 (133). This does not only regulate transcriptional activity but may also regulate on the level of translation. It was shown that Notch1-RBP-J signaling is required for the activity of the MNK1/eIF4E axis, which controls the rapid induction of IRF8 upon TLR4 stimulation on the protein level. Thereby, Notch1 controls the IFR8 dependent expression of M1 macrophage-associated genes like *Il12a* and *Nos2* (44). Syndecan-1 and 4 are subjected to constitutive and inflammation-induced shedding by ADAM17 (134). However, it seems that syndecan-1 plays a more prominent role in infectious diseases, which is already obvious from the huge variety of interaction partners including matrix proteins, proteases, cytokines, and growth factors [for review see (135)]. *P. aeruginosa, S. aureus* and *S. pneumoniae* were shown to induce syndecan-1 shedding correlating with susceptibility and systemic spread (70, 136, 137), and syndecan-1 serves as attachment receptor for HIV-1 (39). It has been reported that the SICD might acts inhibitory during tumor cell migration and that it might be essential for exosomal release (18, 135); however, a specific function in infectious disease has not been reported so far. Similarly, it is obvious from the mentioned studies that E-cadherin plays in important role in acute and chronic infectious diseases and has been recently reviewed (138). The EICD is associated with beta-catenin and α-catenin and thereby linked to the cytoskeleton. The disassembly of this complex upon ectodomain shedding and RIPing releases β-catenin, thereby orchestrating the canonical Wnt signaling pathways as integral component of the host response to infection (139, 140). However, it is important to note that E-cadherin is cleaved not only by ADAM10 and other metalloproteinase but also by bacterial proteases themselves. If these remaining transmembrane fragments are subjected to RIPing is still under investigation (138). Furthermore, the E-cadherin c-terminal fragment 2, produced by PSEN1, was shown to increase the degradation of transmembrane amyloid precursor protein (APP) derivatives, preventing the formation of the AICD and promoting the non-amyloidogenic degradation (141). Many *in vitro* and *in vivo* evidences including the analysis of patients' samples point toward a contribution of infection to the late-onset of Alzheimer disease. A strong association was shown for infection with *Chlamydia pneumoniae* (*C. pneumoniae*) [for review see (142)]. Infection of astrocytes with this intracellular pathogen showed an increase of BACE and PSEN1 expression, whereas the activity of ADAM10 was reduced, resulting in enhanced production of beta-amyloid (143). However, the overall mechanisms how infection contributes to late-onset of Alzheimer disease is still not clear. Apart from its sheddase activity, RIPing of ADAM10 itself has been reported. In macrophages infected with HIV-1, ADAM10, cleavage by ADAM15 resulted in the release of the intracellular domain by RIPing, mediating the above describe support of viral translocation (86, 144). Besides γ-secretase, which belongs to the aspartyl intramembrane proteases, rhomboids, site-2 protease, and Ras-converting enzyme 1 are intramembrane proteases. However, due to their subcellular localization these proteases are not involved in RIPing of ADAM substrates (145). Thus, there are clear evidences that RIPing by γ-secretase plays an important function in infectious diseases; however, little is known about the exact consequences of these processes.

## SARS-COV-2 and COVID-19

There are only a few examples, in which a direct pathogen-ADAM protease-target molecule axis has been established. Besides ADAM10/*S. aureus* (40) and ADAM9/EMCV (25, 26), the severe acute respiratory syndrome coronavirus 2 (SARS-CoV-2) causing COVID-19 is the most prominent example, showing the diverse protective and progressive action of ADAM proteases in infectious diseases. The extracellular domain of angiotensin converting enzyme II (ACE2), a crucial shedding substrate for ADAM17 (146), functions as cellular receptor for the virus' spike (S) protein (147). ACE2 is highly expressed in the lung and myocardium, and it has been speculated that ADAM17 overexpression could play a milestone protective role through mediating shedding of ACE2 leading to downregulation of its surface expression, inhibiting the key entrance of SARS-CoV-2 and further blocking circulation virus particles (71). After ligation of ACE2 with the spike protein, a proteolytic cut of the S glycoprotein mediated by furin is a crucial step for virus entry (148). One mediator regulating furin expression on the transcriptional level is Notch1 (149). Both, ADAM10 and ADAM17 are able to shed Notch1 and thereby activate down-stream signaling processes. However, cleavage occurs context dependent, with ADAM10 being responsible for ligand-induced signaling, whereas ligand-independent signaling requires ADAM17 (150). Ectodomain shedding of Notch1 is followed by γ-secretase cleavage leading to regulation of Notch targeting genes such as furin and enhancement of ADAM10 activity (149). On the other hand, Notch1 has been shown to function as negative regulator of ADAM17 through the transcription of miRNA-145 (151). Thus, inhibition of ADAM10/ADAM17 in intending to block Notch signaling resulting in downregulation of furin expression and its activation circle may serve as an important approach to prevent viral entry and SARS-CoV-2 infection (38). On the other hand, ADAM17 is the main sheddase of TNF-α and further involved in IL-6R shedding (48). Both, IL-6 and TNF-α are important components of the cytokine storm observed in COVID-19 patients (152). With respect to TNF-α, inhibition of ADAM17 as preventive mechanism could be desired. However, the effect on IL-6 signaling would be limited to ADAM17-dependent IL-6R trans-signaling but would not affect the binding of IL-6 to membrane-bound IL-6R or soluble IL-6R derived from alternative mRNA splicing (153, 154). Furthermore, ACE2 depletion on the cell surface is a key pathological feature of SARS-CoV-2 infection, disabling its protective role and consequently resulting in cardiovascular diseases such as atrial fibrillation and heart failure (72). Furthermore, down-regulation of ACE2 severely worsened the outcome in SARS-CoV infection (155), and pharmacological inhibition and gene silencing of ADAM17 showed a markedly decrease of infection in *in vitro* and *in vivo* models (156). Thus, the role of ADAM proteases during SARS-CoV-2 infection is controversy and has to be carefully evaluated for potential treatment options.

## Silent Effects of Infection—Cardiovascular Disease Development

As mentioned above, local infection may cause severe side-effects in other organs or the whole body, such as sepsis formation upon infection with *P. aeruginosa* (157) and the development of hepatocellular carcinoma metastases upon hepatitis B virus infection (158). The most prominent example is in these days the development acute coronary syndromes, arrhythmias, exacerbation of heart failure, thromboembolism, and myocarditis upon SARS-CoV-2 infection, which may be related to the hyper-inflammatory response [for review see (159)]. TNF-α is one of the most essential cytokines that coordinates the host response against intracellular pathogens, including apoptosis for pathogen deposition, upregulation of adhesion molecules on endothelial cell and leukocytes for inflammatory cell recruitment and initiation of the systemic response and the action as negative regulator through the interaction with TNFRs (160, 161). Although this activity is tightly regulated, constantly elevated levels of TNF-α (e.g., chronic infection or cytokine storm) may cause detrimental vascular damage through the induction of endothelial apoptosis, vascular remodeling, pro-coagulatory effects such as enhanced expression of tissue factor, oxidative stress, and recruitment of inflammatory cells while reducing NO production and the number of regenerating stem cells [for review see (162, 163)]. Several studies have shown the direct link between infection and atherosclerosis development, e.g., with *C. pneumonia* and *Pyrophyromonas gingivalis* (164, 165). Based on autoimmune diseases, it is widely discussed if anti-TNF-α therapy could be used as treatment option in cardiovascular disease, but further studies are required for proof of concept (162). It is important to note that such a treatment would again enhance the susceptibility for infection (166). Besides TNF-α, ACE2 plays an essential role in SARS-CoV infection. As a consequence of infection, ACE2 is downregulated on the surface accompanied by enhanced levels of angiotensin II (Ang II) and activation of the renin-angiotensin system (167). It was shown that the conversion of Ang II to Ang 1-7 by ACE2 has cardioprotective function and that the described dysregulation of ACE2 is not unique for SARS-CoV-2, thus prompting toward administration of soluble ACE2 to prevent organ failure (168). Similar divergent functions of Notch activation in cardiovascular damage have been described. On the one hand, Notch exerts a pro-inflammatory action through the polarization of inflammatory cells and the production of IL-6 through the delta-like ligand 4/Notch1 axis (44, 45). On the other hand, it was shown that ADAM10-dependent signaling through Notch1 and Notch4 cleavage controls organ-specific vascular bed formation (169) and could be a therapeutic option to enhance the regenerative capacity of endothelial progenitor cells (170). Both Notch1 signaling and the shedding of E-cadherin were shown to contribute to endothelial malfunction upon *S. aureus* infection (40, 74, 171). Furthermore, infection with *Neisseria meningitidis* resulted in cleavage of the anticoagulant endothelial protein C receptor (EPCR) and impairment of protein C activation, thereby increasing the risk of thrombosis formation (172). Of course, a huge variety of ADAM substrates have been shown to be involved in vascular biology (15). The membrane-bound IL-6R was upregulated on SMCs of patients suffering from pulmonary arterial hypertension (173), and IL-6R trans-signaling critically contributed to pancreatitis-associated lung injury and in infection with *L. monocytogenes* (64, 174). Furthermore, ADAM8 is involved in the development of acute and chronic inflammatory lung diseases, and soluble ADAM8 was shown to be associated with atherosclerosis and myocardial infarction (175). Thus, it is obvious that both ectodomain shedding and RIPing in infection may contribute to the development of cardiovascular diseases. However, which substrates contribute in a time- and context-dependent manner has still to be investigated for the development of effective treatment options.

## Conclusion and Outlook

**Figure 1:**
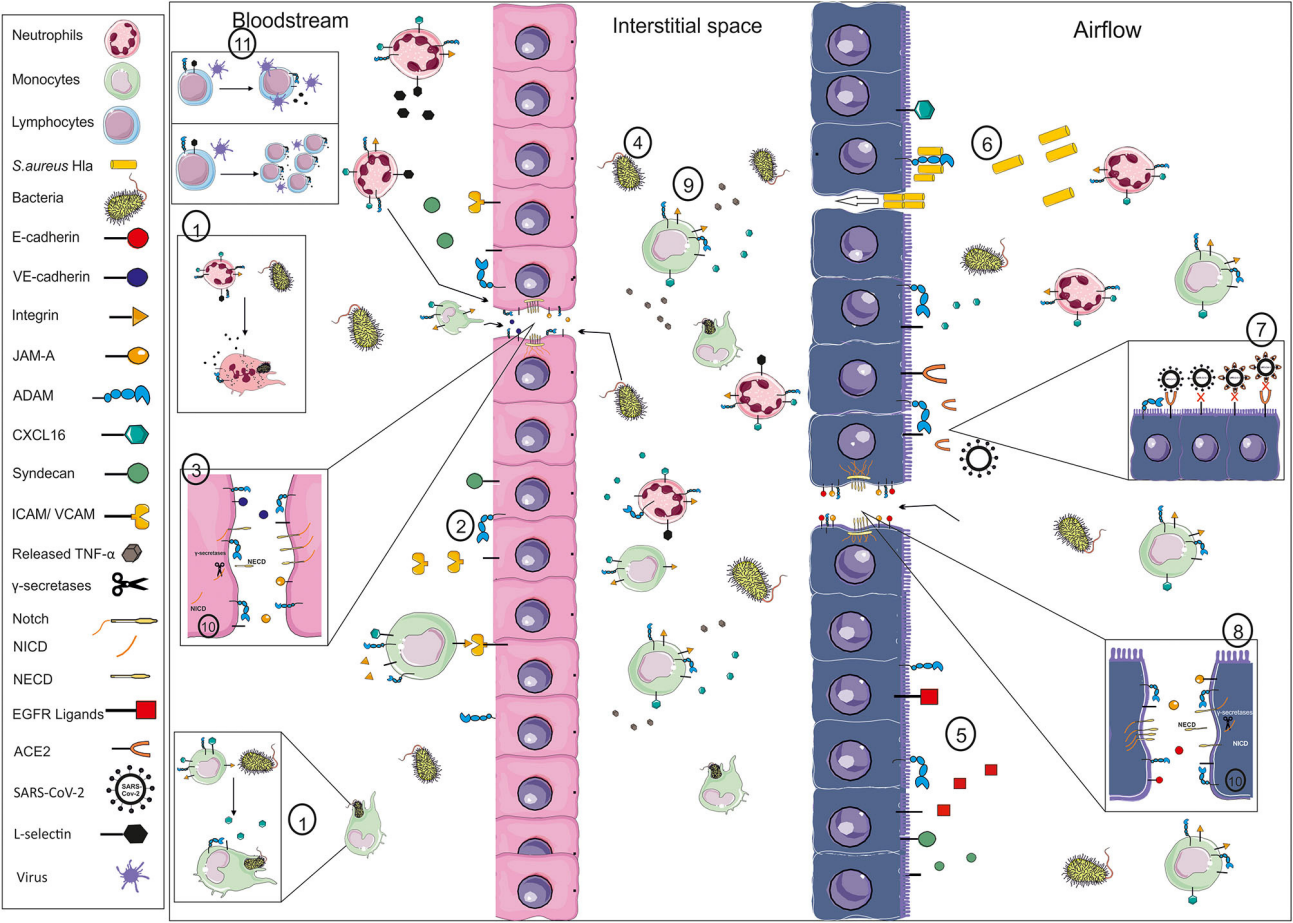
Exemplary illustration of ADAM function in infection. Pathogens like bacteria and their toxins may enter the body as exemplarily shown for the blood stream (left side) and the respiratory system (right side). The first line of defense for systemically entering pathogens is circulating monocytes and neutrophils and the endothelial barrier. ADAM proteases are involved in phagocytosis through, e.g., cleavage of scavenger receptors like CXCL16 or the cleavage of L-selectin (1), in the activation of the endothelium changing the recruitment of inflammatory cells through cleavage of adhesion and junction molecules like ICAM, VCAM, JAM-A and VE-cadherin (2), resulting in gap formation and disturbance of the barrier integrity (3). Thereby, not only inflammatory cells are able to enter the interstitial space but also pathogens (4). Mucus production and the ciliary movement are the natural barrier for pathogens entering via the airflow, which are influenced by the release of EGFR ligands (5). The epithelium expresses several receptors for pathogens, such as ADAM10 in the case of *S. aureus* HIa (6) or ACE2 in the case of SARS-CoV2 (7). Cleavage of ACE2 could limit the viral uptake through lack of receptor and the blocking of circulating virus particles. Pore formation and cleavage of junction molecules like E-cadherin (8) result in crossing of the pathogens and infection of the tissue. The infection is may be cleared by resident or newly recruited leukocytes and a beneficial systemic response, e.g., initiated by TNF-alpha release (9). However, systemic spread of the pathogens, e.g., amplified by the cleavage of syndecans and Notch-1, and enhanced release of pro-inflammatory mediators may cause systemic effects like sepsis or cardiovascular failure. These events are not only orchestrated by α-secretase cleavage but further regulated through RIPing processes mediated by γ-secretases (10). Furthermore, systemic effects may be based on changes in cytotoxic T cell expansion or viral release from central memory T cells (11), both involving L-selectin shedding. These are only a few examples of the ADAMs' impact in infection, which may differ in an organ- and site-specific manner. (VE-cadherin, vascular endothelial cadherin; JAM-A, junctional adhesion molecule A; ADAM, a disintegrin and metalloproteinase; ICAM, intercellular adhesion molecule; VCAM, vascular cell adhesion molecule; TNF, tumor necrosis factor; NICD, Notch intracellular domain; NECD, Notch ectodomain; EGFR, epidermal growth factor; ACE2, angiotensin converting enzyme 2).

ADAM proteases fulfill different functions from pathogen recognition to resolution of the concurrent inflammation and the development of systemic effects (Tables 1, 2, Figure 1). Thereby, they are essential control elements of infectious diseases. However, there a still some limitations with respect to the development of effective ADAM based therapeutic intervention. First, a lot of evidences are based on *in vitro* studies not integrating the divergent functions of substrates in different cell types such as reported for Notch. Second, we did not discuss the complex regulation of ADAM proteases by interaction partners such as tetraspanins for ADAM10 [for review see (176)] and iRhoms for ADAM17 [for review see (177)] or the inhibition by endogenous inhibitors such as tissue inhibitor of metalloproteinase (TIMP) 1 or 3 (37, 178, 179). Of course, dysregulation of these factors may critically contribute to function of ADAM proteases in infectious diseases. Third, we focused on catalytic functions of ADAM proteases with direct evidences from infection studies. However, their general contribution by catalytic and non-catalytic functions to cell migration [for review see (180)] may further influence the defense against pathogens. Fourth, species specific differences in the use of cleavage events as has been reported for IL-6R (181) draw an even more complex view of the ADAM protease web in infection. Nevertheless, this web highlights a huge variety of molecular targets for the treatment of infectious diseases. Targeting ADAM proteases themselves could include inhibition of expression, maturation, or activation, inhibition of the active site by small molecules, application of the inhibitory pro-domain, or inhibition of substrate recognition [for review see (182)]. So far, clinical trials failed due to detrimental side-effects through global changes of ADAM activity and disturbance of tissue homeostasis and developmental processes or lack of efficacy [for review see (12, 13, 23, 183)]. However, several clinical trials are ongoing to address the activation of ADAM10 for the treatment of Alzheimer disease [for review see (184)]. Thus, future studies will have to address ADAM protease functions in a pathogen-, time-, and substrate-specific manner to evaluate specific and efficient treatment options with no or minimal side-effects.

## Author Contributions

AA and DY viewed the literature and wrote the review.

## Conflict of Interest

The authors declare that the research was conducted in the absence of any commercial or financial relationships that could be construed as a potential conflict of interest.
